# Microvascular Complications, Peripheral Artery Disease and Mortality in Patients with Type 2 Diabetes Mellitus, in Two Counties of Southern Lithuania over 13 Years: Analysis Using a Cohort Database of the National Health Insurance

**DOI:** 10.3390/medicina57121380

**Published:** 2021-12-18

**Authors:** Laima Piliponienė, Džilda Veličkienė, Rima Kregždytė

**Affiliations:** 1 Faculty of Public Health, Lithuanian University of Health Sciences, LT-47181 Kaunas, Lithuania; rima.kregzdyte@lsmuni.lt; 2 Institute of Endocrinology, Lithuanian University of Health Sciences, LT-50009 Kaunas, Lithuania; dzilda.velickiene@lsmuni.lt; 3 Neuroscience Institute, Lithuanian University of Health Sciences, LT-50161 Kaunas, Lithuania

**Keywords:** type 2 diabetes mellitus, complications, mortality

## Abstract

*Background and Objectives:* People living with diabetes mellitus are at risk of developing many serious and life-threatening complications. The present study aimed to determine the occurrence of microvascular complications, peripheral artery disease, and mortality in patients with type 2 diabetes mellitus (T2DM), in 2 Lithuanian counties. *Materials and Methods:* The data on residents aged ≥ 18 years, who were diagnosed for the first time in 2004 with uncomplicated T2DM, were obtained from the National Health Insurance Fund database. The occurrence of T2DM microvascular complications, peripheral artery disease, and mortality during the period from 2004 to 2016 were assessed by gender and age groups (<65 and ≥65 years). *Results:* During the 13 years, 46.9% of the patients developed T2DM complications. More men than women developed at least 1 T2DM complication (50.8% vs. 44.8%, *p* = 0.035). The mean time for developing any T2DM complication was 9.2 years. The probability of occurrence of any complication was 0.07 in the second year and increased to 0.59 in the thirteenth year of living with diabetes. Within the 13 years, 38.2% of the patients died. More men (43.1%) than women (35.5%) died during the analysis period (*p* = 0.036). Mortality was higher among older patients (60.7%) than among younger patients (22.2%) (*p* < 0.001). *Conclusions:* The results of this study provide a comprehensive picture of microvascular complications, peripheral artery disease, and mortality among patients with T2DM of two Lithuanian counties. Information about the occurrence of T2DM complications and mortality will assist further studies in estimating the burden of T2DM and in performing economic evaluations of T2DM prevention and treatment in Lithuania.

## 1. Introduction

Diabetes mellitus (DM) is one of the fastest-growing health challenges of the 21st century. People living with DM are at risk of developing many serious and life-threatening complications, leading to an increased need for medical care, reduced quality of life, and undue stress on families [1]. DM and its complications, if not well managed, can lead to frequent hospital admissions and premature death [1]. Globally, DM is among the top 10 causes of death [1]. An estimated 463 million adults, aged 20 to 79, worldwide (9.3% of all adults in this age group) have diabetes [1]. Based on the 2019 estimates, by 2030, a projected 578.4 million, and by 2045, a projected 700.2 million adults, aged 20–79 years, will be living with diabetes [1]. Type 2 diabetes mellitus (T2DM) is the most common type of diabetes, accounting for around 90% of all cases of diabetes [2,3,4].

Persistently high blood glucose levels and insulin resistance cause generalized vascular damage leading to micro- and macro-vascular complications, such as diabetic retinopathy, nephropathy, neuropathy, and cardiovascular diseases, including peripheral artery diseases. The aforementioned complications greatly contribute to morbidity and mortality in both types of DM [5]. Diabetes is one of the leading causes of cardiovascular disease, blindness, kidney failure, and lower-limb amputation [1]. There are no detailed global estimates of diabetes-related complications, but where data are available—mainly from high-income countries—the prevalence and incidence of diabetes-related complications vary considerably among countries [6,7], while data on peripheral artery disease (PAD) are especially scarce. PAD and heart failure are the most common initial manifestations of cardiovascular disease, according to data from nearly 2 million individuals with T2DM and a 5.5-year median follow-up [8]. Subjects with T2DM have a 2- to 3-times higher risk of cardiovascular disease and death. Their life expectancy is estimated to be 8 years shorter than those without diabetes [9].

The burden of DM drains national healthcare budgets, reduces productivity, slows economic growth, causes catastrophic expenditure for vulnerable households, and overwhelms healthcare systems [1]. Along with the increase in prevalence, there comes an inevitably increased economic cost [10]. DM is a serious threat to global health in Lithuania as well: the number of people with DM in Lithuania is increasing, and in 2020, 141,366 people were diagnosed (including 135,039T2DM cases) [11]. The Lithuanian budget expenditure on medicines for T2DM treatment in 2016 was EUR 16.7 million; in 2018, it was EUR 4.75 million for the treatment of T2DM with diabetic neuropathy (DN) [12,13]. The level of well-controlled diabetes is low in Lithuania: less than 30 to 40% of patients with diabetes achieve good diabetes control (expressed as HbA1C equal or inferior to 7%) [14] and thus, we can presume that diabetes complications are prevalent.

In Lithuania, one study has been conducted to analyze mortality risk in people with T2DM. According to the study results, people with T2DM in Lithuania had a 35% higher risk of mortality from all causes. Excess mortality was substantially higher in people who were diagnosed with T2DM at a younger age, died at a younger age, who had a longer diabetes duration, and required treatment with insulin. Women had a higher risk than men in all groups, according to age, time after diagnosis, and therapy [15]. 

To date, studies in Lithuania have examined type 1 diabetes and its complications, but no studies analyzing the detailed development of chronic T2DM complications have been conducted in Lithuania. In addition, there are no studies in Lithuania analyzing the long-term probability of death in newly diagnosed T2DM patients. Therefore, this study aims to determine the occurrence of T2DM microvascular complications, peripheral artery disease, and mortality in patients with T2DM over 13 years, and to compare them by age groups and gender with the official data from the National Health Insurance Fund database. 

## 2. Materials and Methods

The data on the participants, aged 18 years or more and residing in the Kaunas and Marijampolė counties, and who were diagnosed for the first time in 2004 with uncomplicated T2DM, were obtained from the National Health Insurance Fund database. In 2004, 866,132 people lived in the aforementioned counties and accounted for 25.5% of the total population of Lithuania [16]. Analysis was performed using a national health insurance cohort database. This descriptive study assessed the occurrence of chronic microvascular T2DM complications, PAD, and mortality during a period from 2004 to 2016. 

The following T2DM complications were noted, according to the International Statistical Classification of Diseases and Related Health Problems (Tenth Revision, Australian Modification, ICD-10-AM) [17]: T2DM with renal complications (E11.2), T2DM with ophthalmic complications (E11.3), T2DM with neurological complications (E11.4), and diabetic peripheral artery disease (E11.5). The International Statistical Classification of Diseases and Related Health Problems (Tenth Revision, Australian Modification, ICD-10-AM) is used by the Lithuanian health care system. In Lithuania, a diabetes diagnosis is confirmed according to the World Health Organization definition [18]. Complications according to ICD-10-AM coding are provided by the physicians treating the patients based on laboratory measurements or physical examination according to the local rules and guidelines. A diagnosis of diabetic retinopathy is confirmed by an ophthalmologist; depending on the presence or absence of abnormal new vessels, it is classified as non-proliferative (background/pre proliferative) retinopathy or proliferative retinopathy. The diagnosis of DN and nephropathy is confirmed by endocrinologists or general practitioners; the diagnosis of DN is confirmed with screening tests for symmetrical loss of sensation in the legs [19]; diabetic nephropathy is confirmed by an increased urinary albumin excretion (≥30 mg/24 h urine collection) and/ or at least a twice increased urinary albumin to creatinine ratio >30 mg/g (>3 mg/mmol]) in a spot urine sample, persisting longer than 3 months [20]. The etiology of analyzed microvascular complication as diabetic was confirmed by treating physician based on their best judgement and knowledge. The National Health Insurance Fund database, created in 1999, contains demographic data and entries on provided healthcare services, whether ambulatory or hospital-based, and prescriptions of reimbursed pharmacological agents. Based on these data, the provided services are reimbursed, thus health care institutions have the interest to enter all of the data correctly. All of the state-owned and majority of private medical institutions have an obligation to report data of the provided services to the National Health Insurance Fund. The database covers about 99 % hospitalized patients and about 90% outpatient visits [21].

If T2DM complications occurred within one year of the diagnosis of an uncomplicated T2DM, the cases were excluded from the study. Such cases could be considered biased because they usually are confirmed by specialists (endocrinologists, ophthalmologists) and time necessary for the obtaining these consultations sometimes extends to several months. Therefore, the complications might be diagnosed within the next year but actually they are already present at the time of T2DM diagnosis. A total of 108 patients were diagnosed with diabetes complications during the first year and were excluded from the analysis.

Statistical data analysis was carried out using the SPSS (Statistical Package for the Social Sciences) version 20. The cumulative incidence of the developed complications, mortality, probability of complications and death were compared by gender and age groups (patients under and over 65 years old). Cumulative incidences were calculated by dividing number of new complications by the total number of patients with new T2DM diagnosis at the start in 2004. The distribution of data was examined using the Kolmogorov-Smirnov test. Continuous data were compared using the parametric t-test and categorical data with the chi-square test. The time up to the development of complications was assessed by a Kaplan-Meier estimate. The curves were compared using the log-rank (Cox-Mantel) test. The differences between the groups were considered statistically significant when *p* < 0.05. 

## 3. Results

A total of 1044 patients aged ≥18 years were diagnosed with T2DM without any complications for the first time in 2004. There were more women than men (n = 668, 64.0% vs. n = 376, 36.0%), and individuals younger than 65 years accounted for 58.3%. The mean age at T2DM diagnosis was 62 years. The youngest patient was 18.3 years old, and the oldest patient was 100.3 years old. The mean age of the patients in the <65 and ≥65 age groups was 53.8 and 73.5 years, respectively. There was a significant difference in the percentage distribution of women and men by age groups (*p* < 0.001). The baseline characteristics of the study population are shown in Table 1.

**Figure 1:**
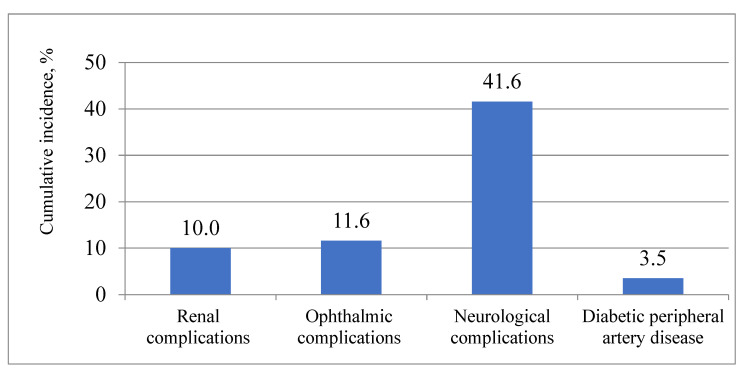
Cumulative incidence of various chronic T2DM complications developed in the overall study population over 13 years.

During the 13 years, 46.9% of the patients developed T2DM complications, from the mentioned complications. Nearly one-third (30.7%) of the patients developed one complication; 12.8%, 2 complications; 3.2%, 3 complications; and 0.2%, 4 complications. More men than women developed at least one T2DM complication (50.8% vs. 44.8%, *p* = 0.035). T2DM complications occurred more frequently in patients who were younger than 65 at the time of diagnosis of T2DM than in those who were 65 and over (55.2% vs. 35.4%, *p* < 0.001). Neurological T2DM complications (E11.4) were the most common over the 13 years. Figure 1 depicts the cumulative incidence of various chronic T2DM complications in the overall study population over the 13 years.

The cumulative incidence of nephropathy (E11.2) and retinopathy (E11.3) significantly differed by gender (Table 2). Retinopathy (E11.3) and peripheral neuropathy (E11.4) occurred more frequently in patients who were younger than 65 at the time of diagnosis of T2DM, than in those 65 and over at the time of diagnosis of T2DM (Table 3).

**Figure 2:**
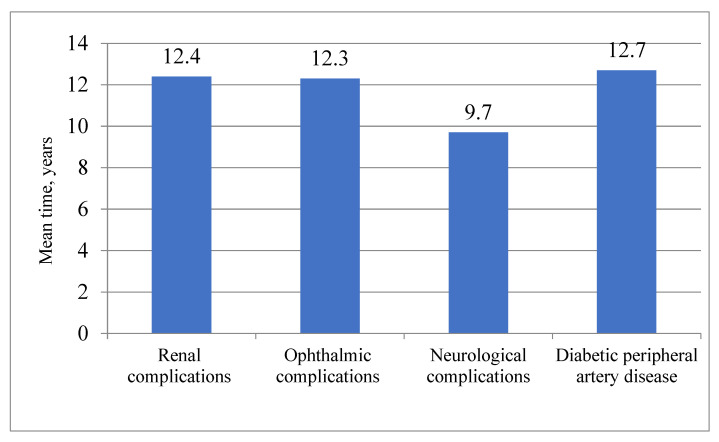
Mean time to the occurrence of various complications from T2DM.

The mean time to develop any T2DM complication was 9.2 years. Figure 2 shows the mean time of the occurrence of various T2DM complications. Men developed T2DM complications earlier than women (after 8.8 vs. 9.4 years, *p* = 0.026). Over the 13 years, any T2DM complication occurred earlier in patients younger than 65 at the time of diagnosis of T2DM, than in those aged 65 and over (8.8 vs. 9.7 years, *p* = 0.005). 

Renal (E11.2) and ophthalmic complications (E11.3) occurred earlier in men than in women. Patients younger than 65 at the time of diagnosis of T2DM developed ophthalmic (E11.3) and neurological complications (E11.4) earlier, compared to those aged 65 and over (Table 4).

**Figure 3:**
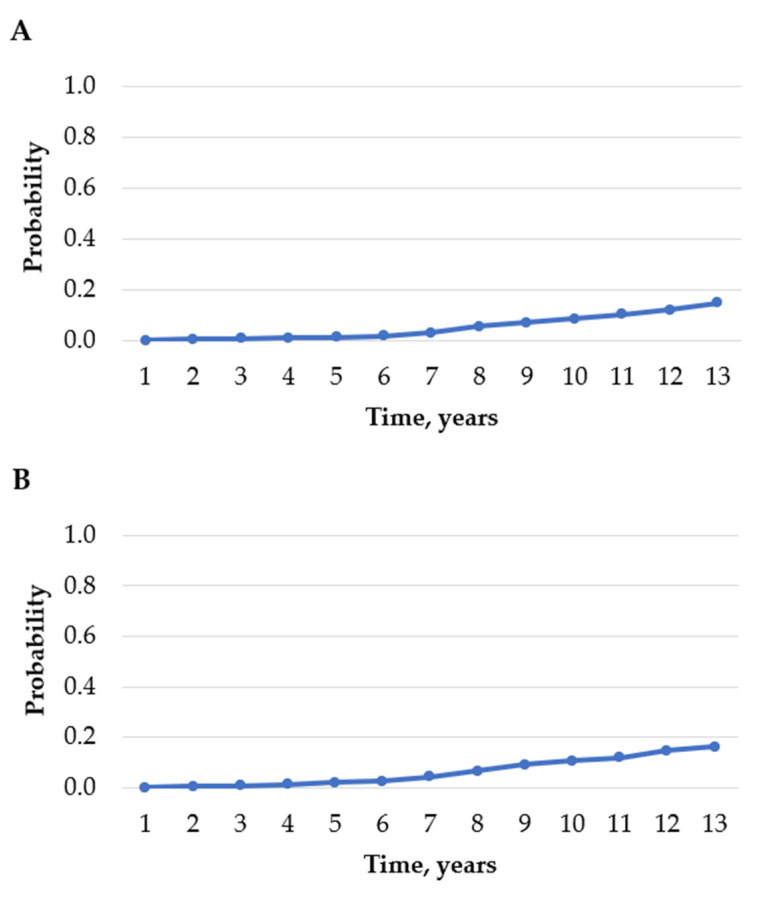

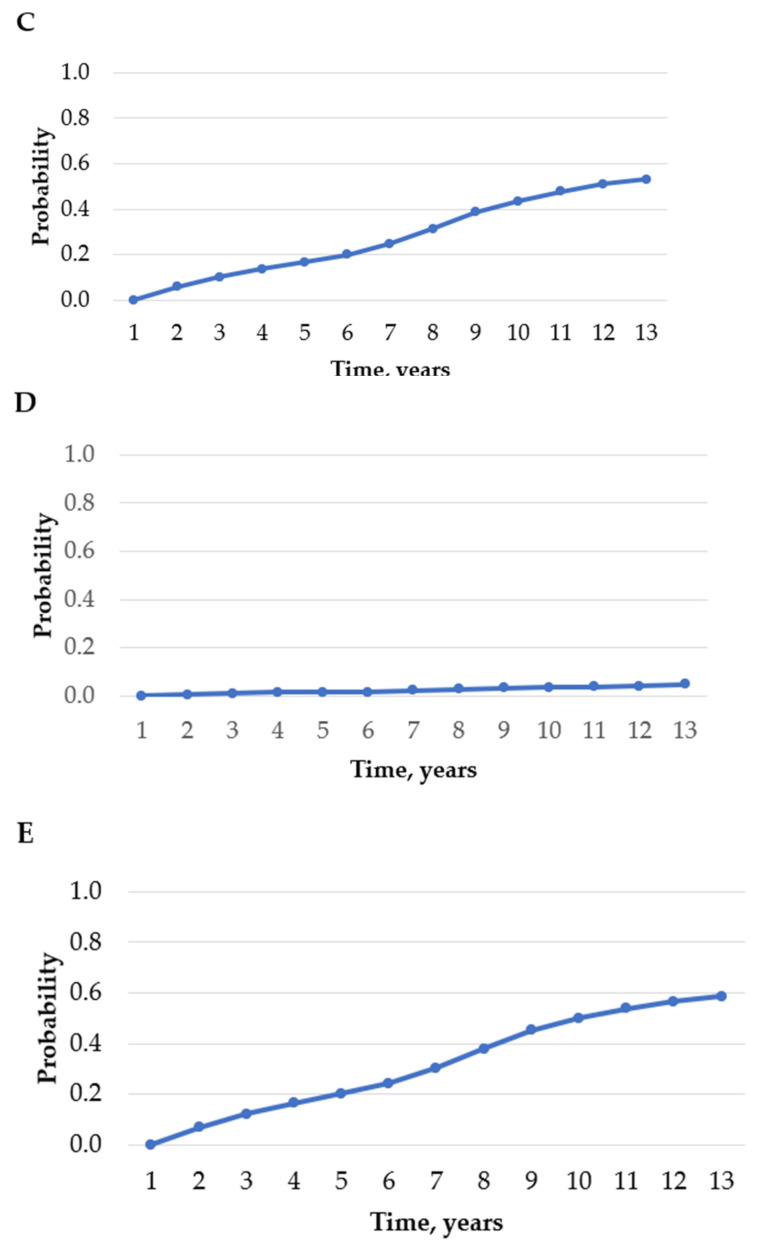
Probability of occurrence of renal complications among patients with T2DM (**A**). Probability of occurrence of ophthalmic complications among patients with T2DM (**B**). Probability of occurrence of neurological complications among patients with T2DM (**C**). Probability of occurrence of diabetic peripheral artery disease among patients with T2DM (**D**). Probability of occurrence of renal, ophthalmic, neurological complications and diabetic peripheral artery disease among patients with T2DM (**E**).

The probability of the occurrence of any T2DM complication was 0.07 in the second year and increased to 0.59 in the thirteenth year of living with diabetes. The probability of developing neurological complications each year for 13 years was the highest, compared to other complications. It increased significantly each year and was 0.06 and 0.53 in the second and thirteenth years, respectively. The probability of developing renal and ophthalmic complications increased as of the seventh year: it was 0.03 and 0.04, in the seventh year, respectively, and 0.15 and 0.16 in the thirteenth year, respectively. The probability of developing diabetic PAD increased marginally during the whole period and it was the lowest in the thirteenth year, compared to other complications. Figure 3 shows the probabilities of occurrence of T2DM complications among patients with T2DM, over 13 years.

During the 13 years, 38.2% of the patients died. More men (43.1%) than women (35.5%) died during analysis period (*p* = 0.036). Mortality was higher among older patients (60.7%) than among younger patients (22.2%) (*p* < 0.001). Within the 13 years, 0.6% of the analyzed patients left the country. 

**Figure 4:**
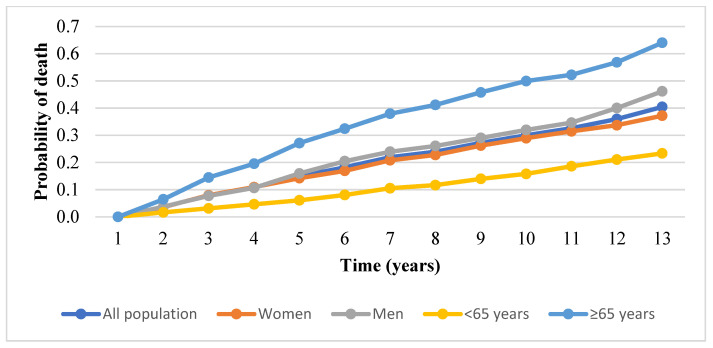
Probability of death among patients with T2DM, over a period of 13 years.

The probability of death was 0.04 in the second year and 0.4 in the thirteenth year. The probability of death in patients with T2DM over 13 years is shown in Figure 4.

## 4. Discussion

The results of this study demonstrate a high cumulative incidence of chronic microvascular complications and peripheral artery disease (PAD) in a cohort of newly diagnosed T2DM in middle-aged persons: almost half of them experienced at least 1 complication over the 13 years, more often men than women, and more frequently patients who were under 65 at the time of diagnosis of T2DM. On average, neurological T2DM complications occurred for the first time after 9 years and were the most common complications for the 13-year follow-up period. These affected more than 40% of patients, whereas all other examined complications developed less frequently and also later, about 12 years after the diabetes mellitus diagnosis: ophthalmological and renal complications were confirmed only in 10% of patients. PAD was the rarest complication and affected only 3% of the study population. 

Numerous studies have analyzed the development of T2DM complications in other countries with a similar population of newly diagnosed T2DM. Our findings are in line with other reports of a high cumulative incidence of neurological complications in T2DM, with a very similar index: about 50% over a very similar follow-up period [22]. Our study identified diabetic neuropathy (DN) as the earliest complication. Several studies observed lower DN indices: researchers in Greece [23] reported a cumulative incidence of DN of 30% during a 7 year-long observation and a Danish study reported a 10% cumulative incidence of DN during a 13 year-long study of the disease [24]. The diagnosis of DN is excessively variable and frequently inaccurate, even when performed by experts of this field [25], and a different approach for diagnosis was used in the Danish study since they used the Michigan Neuropathy Screening Instrument questionnaire, which was completed by participants. The foregoing comments pertain to the explanation of the different results between studies. 

The incidence of diabetic renal complications was twice the amount of ours in other studies. Our study results showed that renal complications, within 13 years of T2DM diagnosis, developed in fewer patients (10% of all patients). In the United Kingdom, the Prospective Diabetes Study (UKPDS) enrolled 5097 individuals, with a median follow-up of 10.4 years, the prevalence of microalbuminuria was 24.9% [26]. The Danish study by Gall et al. followed up 176 normoalbuminuric patients with T2DM, for a median time of 5.8 years, the incidence of diabetic nephropathy was 23% [27]. The differences might be explained by different nephropathy diagnostic criteria, for example, the Gall study only used single urine samples to confirm microalbuminuria. Additionally, both studies were performed in the early nineties of the last century, when the treatment of arterial hypertension and diabetes was not so advanced and did not include medications with renal protection, such as ACE inhibitors, which are a standard part of treatment in the last decades for diabetic patients. Though we were not able to collect information on comorbidities and medications, most diabetics in Lithuania are generally treated for arterial hypertension with ACE inhibitors. It is worth mentioning that the UKPDS study was prospective, and the diagnoses are more precise in studies designed this way, rather than diagnoses obtained from the database.

Diabetic retinopathy (DR) cumulative incidence was also twice as low compared to the UKPDS data, among newly diagnosed T2DM patients. DR after a follow-up of 6 years was diagnosed in 22% of cases [28]. The cumulative incidence of DR was 27.8% among T2DM patients in the Health Improvement Network database (United Kingdom), with 64,983 participants after a 9-year follow-up [29]. Though both mentioned studies included newly diagnosed DM patients, several differences between our study and previous studies of DR need to be considered when comparing results. There exists the possibility of differences between studies because of the techniques used to diagnose eye complications, which could lead to different results. Both studies, similarly to many others of their era (UKPDS study was carried out from 1988 to 1998, and the Health Improvement Network database was compiled from 2000 to 2007), used direct ophthalmoscopy, which is known to have low sensitivity compared to the 2 fields digital retinal photography, which is the accepted method since 2006 in Lithuania and other countries [29]. New trends from the last decade in the standard management of diabetes, dyslipidemia, and hypertension, should also be considered as potential contributors to explain differences between studies. In addition, there might be a systematic bias in our study, such as incomplete compiling of information in the National Health Insurance Fund database or coding problems. Our findings (the cumulative incidence of DR 11.6%) correspond better to a more recent study published in 2013, that used dilated pupils with a slit lamp biomicroscopic examination, with a 90-D handheld magnifier lens and found a cumulative incidence of DR 8.7%, after observation for 4 years [30].

Our findings on the cumulative incidence of PAD are in line with several other reports, as this complication was the rarest of those we examined and was observed only in 3.5% of cases. In UKPDS, after a 6-year follow-up of 5102 individuals, PAD was diagnosed in 2.7% of individuals [31]. In a study conducted with 10,624 T2DM patients from around the world, PAD occurred in 5.8% of patients with a median follow-up of 5 years [32]. The correct diagnosis of PAD also requires special skills and medical equipment, and can thus lead to underdiagnosed PAD, especially in the early stages of our study and the mentioned studies. A report from England from 4 electronic health data sources between 1998 and 2010 found a prevalence of PAD in 16.2% of the 6137 analyzed T2DM patients [8]. It seems that risk factors are equally controlled in many countries, and therefore, this leads to the similar indices. 

Our study detected that at least one T2DM complication developed more frequently and earlier in men than in women and occurred more frequently in patients who were under 65 at the time of diagnosis of T2DM. To the best of our knowledge, we have not found data on gender and age limit at the time of diagnosis being risk factors for diabetes complications and have no clear explanation for this but can speculate that people pay less attention to their health issues at the beginning of the disease and would rather wait until retirement (which is at 65 in Lithuania), which eventually leads to complications. Some evidence exist that diabetes differently damage both micro and macro vessels in men and women. As for microvascular complications, the issue of sex-gender differences is very complex, with many hypotheses explaining the different possibilities, but with no clear evidence for complication differences between genders. The main problem is that it is difficult to differentiate how the common pathogenetic mechanisms of diabetes differently impact the different genders, though it is a well-known fact that diabetic women are at greater risk of cardiovascular complications, compared to non-diabetic people, where the risk for cardiovascular disease is higher in men [33]. 

In the Swedish study by Andersson T et al., during the 24 years of follow-up, 4364 deaths (58.5%) occurred among the 7461 patients with new-onset T2DM. [34]. According to another Swedish study, during 15 years, 52% of the new adult-onset T2DM patients died [35]. In our study, during the 13-year observational period, 38.2% of the patients died. Mortality in our study was lower comparing with the results of other mentioned studies.

Linkeviciute-Ulinskiene D et al. have analyzed the excess risk of mortality among people with T2DM in Lithuania [15]. They found that people with T2DM had a higher mortality risk (standardized mortality ratio (SMR)= 1.35, (95% CI 1.34–1.37)), which was higher in women (SMR= 1.43 (95% CI 1.41–1.45)) than in men (SMR = 1.24 (95% CI 1.22–1.27)), in those who were diagnosed with T2DM at a younger age (SMR = 1.68, 95% (CI 1.60–1.76)) and had diabetes for a longer duration. The risk of mortality increased with increasing time since diabetes diagnosis, with SMRs of 1.09, 1.23, and 1.36 in periods 1–5, 6–10, and >10 years after diagnosis, respectively. According to our study results, at the end of the follow-up, the probability of death was higher in men (0.46) than in women (0.37), and also in those who were diagnosed with T2DM at an older age (0.64). We also determined that the probability of death had increased with increasing time since diabetes diagnosis (0.04 in the second year and 0.4 in the thirteenth year). Due to the different methodology used, the results of the studies cannot be directly compared. Though a retrospective cohort study design was used in both Lithuanian studies, but different T2DM cases were included into studies and different indices were analyzed. Only patients diagnosed at the age of 40 or older and patients who had more than 6 prescriptions for reimbursed glucose-lowering medications were included into the study performed by Linkeviciute-Ulinskiene D et al. The occurrence of complications was not taken into account. We analyzed T2DM cases diagnosed at age of 18 or older without complications at the time of diagnosis. Despite the differences, both studies showed an increase in the death probability or risk to die of diabetes with increasing time since diabetes diagnosis.

Finally, diabetes complications continue to be a major public health issue and additional analyses to evaluate the risk factors associated with the occurrence of not a few but of all diabetic complications, with contemporary diabetes management protocols, are needed to develop effective preventive strategies and treatment. The strength of our study lies in the long follow-up period, the evaluation of the development of all microvascular complications, as well as a full-view presentation of the development of many complications in the 13 years since DM diagnosis, and not only one of the complications as in the majority of other publications. 

This was the first study in Lithuania to evaluate the development of T2DM complications and to provide valuable information about the extent of T2DM chronic complications in Lithuania. It will certainly be an asset for future studies. 

## 5. Limitations

There are limitations to this study regarding the inclusion of the study subjects to be mentioned. Firstly, since the data were collected from the National Health Insurance Fund database, the possibility of bias during data entry and coding processes exists. In addition, we cannot exclude the possibility that not all of the patients were identified as having T2DM complications at the time of diagnosis of T2DM; therefore, we could include the cases with undiagnosed T2DM complications as eligible. Secondly, we did not have access to the data on T2DM control in terms of blood glucose levels (HbA1C), body mass index, arterial blood pressure, smoking habits, and could not perform an analysis of these important factors. Thirdly, the diagnosis of neurological diabetic disorders is quite subjective and over-diagnosis cannot be excluded. Finally, we did not analyze the data on other macrovascular complications, such as myocardial infarct, stroke, etc., as the data on these diagnoses in the National Health Insurance Fund database were entered using different codes, which does not enable us to recognize the patient as diabetic. In addition, the diagnosis of comorbidity of diabetes was not always included by cardiologists and other specialists in the discharge summaries. Due to these unfavorable circumstances, we were not able to determine connections between the above-mentioned complications, T2DM, and mortality.

## 6. Conclusions

Almost half of newly diagnosed T2DM patients experienced at least 1 complication over the 13-year study period, more often men than women. The probability of the occurrence of any T2DM complication increased by 8 times during 13 years. Neurological complications were the most common complications. They occurred faster than other complications. The probability of death increased by 10 times during 13 years from the T2DM diagnosis confirmation. The results of this study give a comprehensive picture of T2DM microvascular complications, peripheral artery disease, and mortality among patients with T2DM, and enable the simulation of the long-term outcomes of T2DM progression. The results of the occurrence of T2DM complications and determined mortality will assist in estimating the burden of T2DM and in performing economic evaluations of T2DM prevention and treatment in Lithuania in future studies.

## Figures and Tables

**Table 1:** Baseline characteristics of the study population.

	Value
Women, *n* (%)	668 (64.0)
<65 years	357 (53.4)
≥65 years	311 (46.6)
Men, *n* (%)	376 (36.0)
<65 years	252 (67.0)
≥65 years	124 (33.0)
Age, mean ± SD, years	
Women	63.6 ± 12.1
Men	59.2 ± 13.1

**Table 2:** Cumulative incidence of chronic T2DM complications developed over 13 years, by gender.

T2DM Complications	Women, *n* (%)	Men, *n* (%)	*p* Value
Renal complications	50 (7.5)	54 (14.4)	<0.001
Ophthalmic complications	63 (9.4)	58 (15.4)	0.001
Neurological complications	266 (39.8)	168 (44.7)	0.076
Diabetic peripheral artery disease	21 (3.1)	16 (4.3)	0.300

**Table 3:** Cumulative incidence of chronic T2DM complications developed over 13 years, by age groups.

T2DM Complications	<65 Years, *n* (%)	≥65 Years, *n* (%)	*p* Value
Renal complications	72 (11.8)	32 (7.4)	0.693
Ophthalmic complications	97 (15.9)	24 (5.5)	0.002
Neurological complications	303 (49.8)	131 (30.1)	0.005
Diabetic peripheral artery disease	22 (3.6)	15 (3.4)	0.438

**Table 4:** Time to the occurrence of T2DM complications, by gender and age groups.

T2DM Complications	Time to the Occurrence of T2DM Complications, Years		*p* Value	Time to the Occurrence of T2DM Complications, Years		*p* Value
	Women	Men		<65 Years	≥65 Years	
Renal complications	12.5 (12.4–12.7)	12.2 (12.0–12.4)	<0.001	12.4 (12.2–12.5)	12.5 (12.3–12.7)	0.693
Ophthalmic complications	12.4 (12.3–12.6)	12.0 (11.7–12.2)	0.001	12.1 (12.0–12.3)	12.5 (12.3–12.7)	0.002
Neurological complications	9.8 (9.5–10.2)	9.4 (9.0–9.9)	0.076	9.4 (9.1–9.7)	10.2 (9.8–10.6)	0.005
Diabetic peripheral artery disease	12.7 (12.6–12.9)	12.7 (12.5–12.8)	0.300	12.8 (12.7–12.9)	12.6 (12.5–12.8)	0.438

Values are mean (95% confidence interval).

## Data Availability

The data presented in this study are available on request from the corresponding author.
